# Progress in Using Circulating Tumor Cell Information to Improve Metastatic Breast Cancer Therapy

**DOI:** 10.1155/2013/702732

**Published:** 2013-03-25

**Authors:** Jose Alemar, Eric R. Schuur

**Affiliations:** ^1^Florida Cancer Specialists, 100 Highland Avenue, Largo, FL 33770, USA; ^2^VMWA LLC, 2493 Waverley Street, Palo Alto, CA 94301, USA; ^3^The Jon Block Group, 2210 Jackson Street, Suite 401, San Francisco, CA 94115, USA

## Abstract

Circulating tumor cells (CTCs) were discovered nearly 150 years ago but have only recently been recognized as a feature of most solid tumors due to their extremely low concentration in the peripheral circulation. Several technologies have been developed to isolate and analyze CTCs, which can now be routinely accessed for clinical information. The most mature of these (the CELLSEARCH system) uses immunomagnetic selection of epithelial cell adhesion molecule to isolate CTCs for analysis. Studies using this system have demonstrated that categorization of patients into high and low CTC groups using a validated decision point is prognostic in patients with metastatic breast, colorectal, or prostate cancer. Initial attempts to use CTC counts to guide therapeutic decisions appeared to yield positive results and key concepts in clinical application of CTC information, including the CTC cutoff, predictive value in disease subtypes, and comparison to current evaluation methods, have been demonstrated. Clinical studies of the impact of CTC counts in routine clinical practice are ongoing; however, recent published evidence on the clinical use of CTCs in metastatic breast cancer continues to support these concepts, and experience in the community oncology setting also suggests that CTC enumeration can be useful for therapy management.

## 1. Introduction

In the United States nearly 40,000 deaths are attributed to metastatic breast cancer (MBC) annually [1]. Once breast cancer has metastasized it is usually fatal [2]; however, intensive research into the causes of breast cancer and the development of an array of effective targeted therapeutics has improved survival for these women. Appropriate management of treatment is important to make best use of these new therapeutics. Circulating tumor cell (CTC) analysis is a promising tool to address the need for better disease management, potentially improving both survival and quality of life for MBC patients.

## 2. History and Initial Clinical Application

CTCs represent the hematogenous phase of metastasis [3]. They were initially observed over 150 years ago as a leukemia-like manifestation of cancer [4] with very high concentrations of malignant cells in the peripheral circulation. Most instances of metastatic solid tumors are not accompanied by high concentrations of CTCs, so these cells were not a focus of tumor biology research or clinical application until contemporary studies demonstrated the frequent presence of CTCs in patients with solid tumors, albeit at very low concentrations [5–7]. Importantly, CTCs are essentially never found in individuals without malignant disease [8]. The development of methods to reliably detect CTCs in the peripheral circulation has enabled the study of their behavior and has facilitated their clinical use. Of the technologies that have been developed to isolate and enumerate CTCs, immunomagnetic isolation using cell surface epithelial cell adhesion molecule (EpCAM) antibodies, followed by semiautomated fluorescence microscopy analysis has been most widely used [9]. In 2004 the CELLSEARCH System (Veridex, Raritan, NJ, USA), which is based on this approach, was cleared by the USA Food and Drug Administration as an aid in monitoring patients with MBC [8, 10].

The clinical relevance of CTCs was first demonstrated in a landmark study from Cristofanilli and colleagues in patients with progressive MBC on palliative therapy [11]. In that study, the authors showed that classification of patients based on a cutoff of 5 CTCs per 7.5 mL of blood using the CELLSEARCH System informed survival prognosis. This result was subsequently confirmed in patients with newly diagnosed MBC starting first-line therapy [12]. The prognostic properties of CTCs were shown to be robust during therapy by Hayes and colleagues [13]. Budd et al. [14] reported that comparison of CTC enumeration to traditional radiologic assessment as measures of disease status in MBC showed that CTC assessment was a more reproducible indicator of disease status that could be measured earlier in the course of treatment. Similar results have been published demonstrating the relationship of CTC enumeration to survival in prostate, colorectal, and lung cancers and other malignancies (e.g., [15–17]).

**Figure 1:**
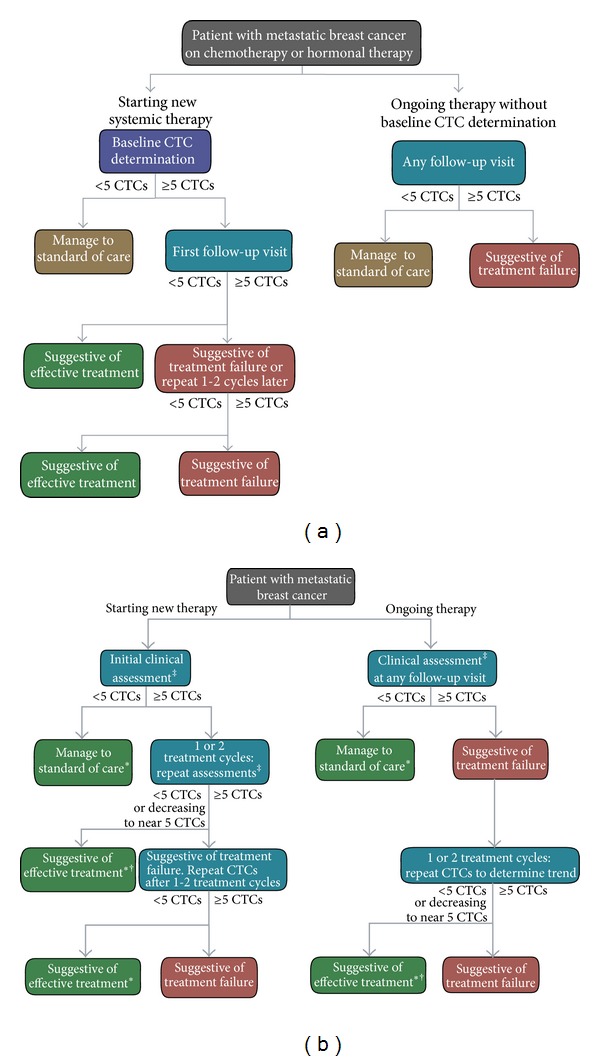
Algorithms for incorporating CTC measurements into clinical decision making. (a) Beveridge algorithm [18] for management of metastatic breast cancer using circulating tumor cell information. (b) An updated algorithm incorporating CTC information in the monitoring strategy. Additional time may be added before a second CTC assessment for patients starting new systemic therapy for CTC counts to stabilize. Declining CTC counts suggest treatment response but require confirmation subsequently. The interval between follow-up CTC counts considers clinical behavior of the tumor. ^‡^Clinical assessment may include imaging studies, physical examination, histology, CTC enumeration, and serum tumor marker determination. *Disease status may be monitored using CTC counts every 1–3 months, with imaging studies performed if clinical evidence of progression is observed. ^†^Repeat CTC assessment after an additional 1-2 cycles of therapy to verify that CTC counts are <5 per 7.5 mL; CTC counts that remain ≥5 upon retesting suggest treatment failure.

The fatal nature of MBC, the role of CTCs in metastasis, their prognostic value, and the deficiencies of imaging and existing biomarkers for monitoring disease progression created a motivating environment to use CTC enumeration to guide clinical decision making in MBC. In 2007 Beveridge [18] reported the use of serial CTC measurement using the CELLSEARCH System to guide therapeutic intervention in a community-based oncology practice. In that work, 50 patients with MBC had CTC measurements at baseline and at subsequent monthly intervals during treatment, along with radiographic studies and CA 27.29 biomarker determinations. A treatment algorithm was developed and implemented with a goal of managing therapy to achieve <5 CTC per 7.5 mL (Figure 1(a)). Continual monitoring in a community practice setting was reported to be useful as a leading indicator of disease course in the context of total evaluation of the patient. 

Below, we review evidence from the literature on the application of CTC enumeration using the CELLSEARCH System in MBC. We then discuss the current clinical applications of CTC enumeration for monitoring MBC in the community setting. The studies reviewed are limited to those reporting results in MBC using the CELLSEARCH system, since clinical experience is most mature in this setting.

## 3. Integration of CTC Measurement with MBC Therapies

The concepts that underlie integration of CTC enumeration with MBC treatment in the model represented by the Beveridge algorithm include evidence of prognostic utility, applicability across a range of disease phenotypes and treatments, and comparability to currently used methods of disease assessment (Table 1).

### 3.1. Validation of the 5 CTC Cutoffs for Categorization of ****Survival Prognosis at Baseline and during Treatment

The CTC count is a continuous variable; however, Cristofanilli and colleagues dichotomized it to facilitate clinical application [11]. The decision point of ≥5 CTC per 7.5 mL of peripheral blood for the CELLSEARCH System was determined by maximizing the Cox proportional hazards ratio (HR) for progression-free survival (PFS) between the group of patients with CTC levels below the cutoff value and those at or above the cutoff value. The HR reached a plateau at 5 CTC per 7.5 mL. To date, most of the studies of CTC prognosis using the CELLSEARCH System for enumeration have used the ≥5 CTC per 7.5 mL cutoff for categorization.

Four studies since 2007 have addressed the validity of the prognostic categorization of patients using this decision point at baseline [19–22]. Botteri and colleagues evaluated the appropriate cutoff value for categorizing PFS in a series of 80 patients with MBC starting second- or third-line therapies [19]. For prediction of both PFS and overall survival (OS) a decision point of 5 CTC/7.5 mL was determined to be optimal. This result was confirmed by others for patients with progressive disease [20] or newly diagnosed MBC [21] using similar methodology to Botteri et al. Using a Wald test, Bidard et al. calculated that the maximum probability of time to progression occurred at a slightly lower threshold value of 3 CTC in bevacizumab and chemotherapy-treated MBC patients [22].

CTC measurement should be ideal for monitoring disease status because of ease of access for serial measurements. Hayes et al. [13], as well as other early publications using the CELLSEARCH System [11, 14], tested the prognostic value of CTC measurement during therapy, demonstrating that CTC counts during ongoing therapy were significantly associated with prognosis. Additional studies since 2007 have demonstrated the prognostic value of CTC counts in patients with MBC at initiation of a new line of therapy [23–28] and during ongoing therapy [25–31], confirming that CTC values obtained during treatment have essentially the same prognostic value for PFS and OS as baseline measurements and may be useful for monitoring disease status.

Within these two prognostic categories (≥5 CTC and <5 CTC), there is evidence of additional prognostic information. A small proportion of patients have extremely high levels of CTCs (>100 CTC per 7.5 mL); this subgroup is characterized by very short survival [32]. Conversely, approximately 1/3 of MBC patients do not have detectable CTC in their peripheral circulation, which constitutes a positive prognostic factor relative to patients with ≥1 CTC at baseline [33] and during treatment [21].

A recently published meta-analysis examined the prognostic value of CTC measurement in both early stage breast cancer and MBC both at baseline and during treatment [34]. The pooled HR from included studies showed that CTCs were associated with significantly increased risks of disease progression and death, confirming the robust prognostic value of CTC enumeration.

Finally, two currently ongoing clinical trials, SWOG S0500 from the Southwestern Oncology Group and CIRCEO-1 at the Institut Curie, have as their objective to assess the impact of CTC guidance of treatment decisions on patient outcomes. In both the SWOG S0500 trial and the CIRCEO-1 trial the choice of therapy is contingent on measured CTC levels; the relationship of changes in survival to use of CTC counts will be reported.

**Status**

For MBC, use of the ≥5 CTC per 7.5 mL cutoff for categorization of prognosis is supported for patient management. Recent results suggest that additional prognostic categories may be useful.

### 3.2. Modulation of CTC Prognostic Value by Type of Therapy and Disease Subtype

Critical to the application of CTC information to disease monitoring will be an understanding of the influence of disease subtype or type of therapy on CTC behavior. The studies that established the clinical utility of CTC enumeration [11–14] did not directly assess the effect, if any, of the type of therapy (small molecule, monoclonal antibody, radiation, etc.) on the subsequent prognostic value of CTC measurement. The prognostic value of CTC enumeration has consistently been shown to be independent of hormone or chemotherapy. There have been two reports in which targeted therapy of overexpressed HER2 gene product, epidermal growth factor receptor, using either trastuzumab or lapatinib in combination with chemotherapy in MBC appeared to modify the predictive value of CTC counts, possibly due to preferential benefit to the worse prognosis group [24, 35]. An additional report described lack of prognostic value of CTC during therapy in patients treated with bevacizumab [22]. However, other studies have examined chemotherapy alone [29] or in combination with monoclonal antibody components [36, 37] and have not observed an impact of therapy type on CTC prognosis.

Some of these same foundational studies also showed that CTCs were shed into the circulation irrespective of hormone receptor or HER2 status [11, 12]. Subsequently, CTCs have been isolated from peripheral blood of patients with hormone receptor positive, HER2 overexpressing and triple negative tumors [24, 35, 36, 38–40]. Reports that have examined the relationship between MBC molecular subtype and CTC predictive value for survival have not demonstrated an impact of molecular subtype on prognostic value of CTC counts [24, 35, 36, 40], although two possible exceptions merit mention. One study [41] reported that average CTC counts were lower in inflammatory breast cancer (IBC) than in other types of MBC. Kaplan-Meier survival estimates were not significantly different for IBC patients with ≥5 CTCs and those with <5 CTCs. The study was small, so the relationship of CTC levels to IBC prognosis merits additional research to reach a definitive answer. A second study [42] suggested that MBC characterized by exclusively bone metastasis may have elevated CTC counts compared to other forms of MBC.

**Status**

Therapy type does not appear to influence CTC prognostic value, although monoclonal antibody therapies may preferentially benefit patients with a worse prognosis based on CTC levels. All molecular subtypes of MBC appear to shed similar levels of CTCs as detected by the CELLSEARCH System. Initial reports indicate that molecular subtype does not affect the prognostic value of CTC enumeration.

### 3.3. Integration of CTC Information with Imaging and Serum Biomarkers

Current practice is to use radiologic imaging and serum tumor markers, in conjunction with clinical assessment, to monitor disease status. The initial report from Budd and colleagues [14] described the relationship between radiologic evaluation and CTC assessment. In comparing radiologic progression to CTC levels, the authors concluded that CTC levels were less variable, highly reproducible, and better correlated with prognosis than imaging studies. Since that publication, several studies have compared CTCs to traditional radiologic imaging [20, 25]. Liu et al. [25] showed a strong correlation between CTC results and radiographic disease progression in MBC. The authors concluded that their data supported the clinical utility of serial CTC enumeration in conjunction with standard radiographic imaging to monitor disease status and treatment efficacy. CTC enumeration has also been compared to imaging with fluorodeoxyglucose positron emission tomography/computed tomography (FDG-PET/CT) [30, 43]. Serial radiographic imaging and FDG-PET/CT were shown to correlate with CTC levels, with CTC counts having the advantages of higher precision, better responsiveness to disease state changes, and predictive capability. 

A potential limitation to CTC enumeration is an apparent lack of sensitivity [30, 43]. Nevertheless, these studies concluded that CTC measurement is valuable because of its speed, high specificity, and low variability and suggested continued use of imaging in patients without detectable CTCs [30, 43]. However, as noted previously, the absence of CTCs is in itself a relative positive prognostic factor that should be considered along with imaging results.

Sequential assessments of serum tumor markers, such as CA 27.29 and CA 15-3, are often used in conjunction with imaging and clinical evaluation to monitor therapy response. The comparative performance of CTC enumeration to serum tumor markers has also been reported [18, 23, 31, 36]. Cristofanilli et al. [36] reported that CA 27.29 was prognostic of survival when evaluated in a univariate analysis, as was CTC count but that in a multivariate model including both factors, only CTC count remained significant as a predictor of survival. In his prospective study, Beveridge [18] compared the performance of CTC and CA 27.29 for monitoring response. It was reported that CTCs had a high specificity (89%) and moderate sensitivity (70%) to detect radiologic disease progression, while CA 27.29 had a high sensitivity (85%) and a low specificity (31%) in this case series. Beveridge also found that changes in CTC, but not CA 27.29, predicted PFS. Bidard et al. [23] evaluated CTC count, CA 15-3, carcinoembryonic antigen (CEA), lactate dehydrogenase (LDH), CYFRA21-1, and alkaline phosphatase (ALP) as survival predictors and concluded that these measures had globally similar performance. Hartkopf and colleagues [31] compared CA 15-3 concentrations during therapy to CTC counts and found that they were significantly related to each other as well as to OS. Although serum tumor markers and CTC counts share some properties, differences between these measures are apparent. Serum tumor markers can become elevated in nonmalignant disease states, when patients are experiencing a response to treatment, and may respond slowly to changes in disease status. In contrast, CTCs have high specificity for MBC and respond promptly to changes in disease state [8, 14, 44].

Comparison of CTC enumeration to imaging [12, 14] and serum tumor marker measurement [18, 36] shows that CTC level is an independent prognostic factor for survival in MBC. In turn, this suggests that CTC information reflects some aspect of tumor biology not interrogated by the other tools.

**Status**

CTC counts correlate with imaging assessment and serum tumor marker determination but may have specificity and ease-of-use advantages. CTC counts, imaging, and serum tumor markers may have complimentary roles in monitoring disease status [45].

### 3.4. Incorporating CTC Information into MBC Treatment Strategies

The studies summarized above characterized the properties of CTCs with respect to disease state. The ultimate goal for clinical application is to integrate CTC information into disease management strategies. Although the CELLSEARCH System has received FDA clearance for use in MBC and is being used in the community setting to help monitor MBC patients, few studies have been published describing the impact of CTC information on patient care. As discussed by Bednarz-Knoll et al. [46] and Beveridge [18], the clinical objective is to maintain CTC concentrations below 5 per 7.5 mL, although decreasing CTC concentrations from baseline/pretreatment to the first in-treatment assessment can be considered a positive indication of effective therapy. The ultimate clinical goal is to achieve and maintain CTC counts of <5 per 7.5 mL at subsequent CTC assessments [18, 46]. Both of these criteria have support from the studies described above. The additional studies described below more tightly integrate CTC information into personalized disease management.

Wang et al. [44] used measurement of phosphorylated gamma-H2AX in CTCs to monitor therapy of heavily pre-treated MBC patients with progressive disease in a phase I trial of investigational DNA damaging therapeutic agents. The level of nuclear phosphorylated gamma-H2AX is proportional to the number of DNA double-strand breaks caused by DNA damaging agents. The level of nuclear gamma-H2AX was assayed in CTCs isolated from serial blood samples during treatment on the study protocol. The study results showed that gamma-H2AX levels in CTCs correlated with exposure to DNA damaging agents, which suggests that this approach will enable monitoring of therapeutic effectiveness and adjustment at the cellular level.

One case study [47] and one case series [32] describing treatment strategies that include CTC monitoring have been published.

The single case report of a patient with progressive metastatic inflammatory breast cancer in which disease progress was monitored using CTC counts, in conjunction with other biomarkers, was published by Liu and colleagues [47]. CTC concentration and epidermal growth factor receptor status were monitored, along with the serum levels of CA 15-3. Blood samples were taken at baseline and every three weeks. Initiation of therapy resulted in a dramatic remission and a reduction in CTC count to zero at 9 weeks of therapy. CTC counts began to rise again at the 18-week measurement, followed by CA 15-3 three weeks later.

Interestingly, there has been just a single report since Beveridge's article that describes a treatment regimen incorporating CTC enumeration via the CELLSEARCH System in the clinical decision making process for patients with MBC. Graham and colleagues reported on results from a community practice case series where CTC monitoring was used in conjunction with other biomarkers to guide treatment decisions [32]. CTC levels that reached 5 CTC per 7.5 mL or more during treatment suggested a decision to use more aggressive therapy, accounting for patient preference and other clinical factors. Reduced CTC levels supported use of lower morbidity regimens in the subsequent round of treatment. Analysis of disease-specific survival for patients according to the highest level of CTC observed suggested that reduced CTC levels are associated with improved survival.

## 4. Discussion

The literature provides ample support for the concepts on which the Beveridge algorithm was originally based. The decision point of ≥5 CTC per 7.5 mL blood in MBC is supported both at baseline and during ongoing therapy by contemporary studies. Additional granularity of prognostic categories may be possible. The molecular subtype of MBC (except possibly IBC) and, generally, type of therapy do not affect use of CTC information. Finally, CTC counts correlate with radiologic disease progression and with serum tumor marker levels but provide additional useful information.

Based on the growing body of evidence supporting clinical use of CTC analysis, some community-based practices are moving toward routine use of CTC information to facilitate monitoring of disease status from a simple venipuncture. The evidence discussed above suggests that the Beveridge algorithm, used in conjunction with clinical judgment, conventional imaging studies, and tumor biomarkers is a valid guide to use CTC information for MBC therapy.

Figure 1(a) shows the Beveridge algorithm, as originally described [18]. Figure 1(b) shows an updated algorithm that incorporates CTC information in the monitoring strategy. In this model, patients with newly diagnosed MBC starting systemic therapy undergo CTC testing, in addition to imaging, physical exam, and serum tumor marker measurement as part of their clinical assessment (Figure 1(b), left). Patients with CTC counts below the decision level of 5 CTC can be managed to standard of care for their disease, using periodic CTC measurement to monitor disease status. Imaging studies are ordered when clinically indicated. Measured levels of CTCs at or above the decision point suggest that more careful monitoring of disease state is warranted. CTC counts are repeated 1-2 cycles later to allow for CTC levels to stabilize before considering therapy effectiveness. If the CTC count is <5 at the follow-up assessment, the patients are managed to the standard of care, and disease status is monitored with periodic CTC counts, with imaging performed when clinically indicated. If the CTC count is ≥5, treatment failure is possible and new therapeutic options may be considered if the elevated CTC counts persist. In addition to the decision point of 5 CTCs per 7.5 mL of blood, the direction and magnitude of change in CTC levels are important to consider. A decreasing CTC count that is approaching the decision point value might be considered evidence of effective treatment, although the ultimate goal remains a CTC count below 5. This approach is consistent with the results from Coumans and colleagues, which demonstrate that patients with an unfavorable CTC level at baseline whose CTC level falls during treatment to favorable levels survive longer than those with CTC levels that do not decrease [48]. The authors conclude that the cutoff of 5 CTCs is appropriate and that achieving a CTC level below this at 6 to 8 weeks posttreatment is the best indicator of treatment success.

Patients already receiving therapy for MBC, but without a CTC count prior to initiation of therapy can have a CTC count at the time of any clinical assessment to establish a reference CTC level for comparison (Figure 1(b), right). Elevated CTC counts suggest re-testing after a period of continued therapy to establish a trend to guide subsequent treatment decisions. CTC counts below 5 or decreasing to near 5 suggest effective treatment that can be monitored with CTC counts as described previously.

Our experience suggests considering clinical context, including the clinical behavior of the individual's tumor, when deciding how often to measure CTC levels after baseline in a monitoring strategy. For example, estrogen receptor positive MBC with bone-predominant metastases is often a disease with an indolent course compared to MBC with visceral metastases [49]. Favorable CTC counts (0 to 4 CTC per 7.5 mL) in patients with indolent tumors can be placed on a schedule to measure CTC every 2-3 months. Patients with CTC levels at or above 5 or with visceral metastases are monitored monthly with CTC counts.

We have also found serial monitoring of CTC levels as described previously to be useful in assessing patient prognosis where other assessments may not give a clear answer. This is because CTC measurements provide additional information beyond that provided by other assessments; CTC measurements capture information about the biology of the tumor [14, 36, 50] meaning changes in CTC levels may reveal changes in disease status that are not apparent by other assessments. For example, imaging studies provide information on location, volume, and metabolic activity of the tumor; however, response to therapy on imaging does not always correlate well with survival [14] and may reveal a mixed response to treatment [51]. Furthermore, not all metastatic nodules visible to imaging will impact the prognosis of the patient [51]. Likewise, serum tumor markers typically track tumor burden but may rise spuriously. These features of standard assessments can lead to conflicts with clinical judgment that CTC information can help resolve. To illustrate, imaging may reveal an enlarged pulmonary nodule, yet the clinical presentation may not be poor. If the CTC level is favorable, then prognosis is favorable, and we may recommend continuation of the current therapy. Conversely, elevated CTC counts in the face of stable imaging or serum tumor markers suggest poorer prognosis and might suggest that the current therapy may not be effective. This may lead us to alter therapy before changes are evident on imaging or in serum tumor marker levels. Therefore, use of CTC counts to monitor disease status can reduce reliance on imaging thus sparing the patient exposure to ionizing radiation and reducing costs, can speed transition to potentially more effective treatment, and can potentially improve quality of life.

The Beveridge algorithm, as originally published, is supported remarkably well by the more recent literature. However, clinical experience and suggestions in the literature indicate that certain therapeutic situations or clinical features of MBC, such as bone metastasis-predominant MBC or trastuzumab therapy, may influence how CTC counts are used. Additional research is warranted to refine our understanding in these situations. Documenting CTC counts in other clinical situations that are not addressable using clinical trials will add to body of knowledge on how best to use CTC-based information to better manage cancer. For example, CTC counts may be informative in situations of mixed responses on imaging or appearance of new metastatic foci that are not likely to affect the survival of the patient, situations that probably will not be subjected to study in clinical trials. Capture of this information in case reports, along with documentation and sharing of routine clinical experience, will add clarity to clinical use of CTC information.

Beyond enumeration, an advantage to CTCs relative to imaging and serum tumor markers is the access they may grant to molecular information about the tumor. For example, CTCs may provide a more easily accessible tissue source for evaluation of biomarkers, such as HER2 [52, 53] and multidrug resistance proteins [54]. A great deal more remains to be learned about the biology of CTCs in breast cancer. Appreciation is growing for the complexity of breast and other cancers and the heterogeneity of the disease, even within the individual patient. Information from CTCs may be critical to facilitate access to personalized medicine for patients with cancer in the future.

## Figures and Tables

**Table 1:** Key concepts underlying the algorithm for management of metastatic breast cancer using circulating tumor cell information.

Key algorithm parameters	
(1) Validation of the 5 CTC cutoffs for categorization survival prognosis at baseline and during treatment.	
(2) Modulation of CTC prognostic value by type of therapy and disease subtype.	
(3) Integration of CTC information with traditional imaging and serum biomarkers.	
